# The Etiology of Chest Pain in Children Admitted to Cardiology Clinics and the Use Echocardiography to Screen for Cardiac Chest Pain in Children

**DOI:** 10.3389/fped.2022.882022

**Published:** 2022-05-17

**Authors:** Li Chen, Hongzhou Duan, Gang Li, Xiaoyan Li

**Affiliations:** ^1^Department of Pediatric Cardiology, Beijing Anzhen Hospital, Capital Medical University, Beijing, China; ^2^Department of Neurosurgery, Peking University First Hospital, Beijing, China; ^3^Department of Pediatric Cardiology Surgery, Beijing Anzhen Hospital, Capital Medical University, Beijing, China; ^4^Beijing Institute of Heart, Lung and Blood Vessel Diseases, Capital Medical University, Beijing, China

**Keywords:** chest pain, children, diagnostic value, echocardiography, pediatric cardiology clinic

## Abstract

**Aims:**

Chest pain is a common disease in children. Most cardiac specialists use echocardiography to evaluate the etiology of and screen for children’s cardiac chest pain. We analyzed the etiology and echocardiography results of children with chest pain in pediatric cardiology clinics, clarified the disease spectrum and evaluated the diagnostic value of echocardiography in screening cardiac chest pain in children.

**Methods and Results:**

The clinical data of children with chest pain aged younger than 18 years who admitted to the pediatric cardiology clinic of Beijing Anzhen Hospital between 2005 and 2019 were analyzed. The patients were divided into three groups, including the preschool group, the school-age group and the adolescent group. Total 3,477 children were enrolled in this study. 232 (6.7%) patients were caused by cardiac diseases and chest pain was of non-cardiac origin in 3,245 patients (93.3%). The incidence of non-cardiac chest pain in the adolescent group was significantly lower than the other two groups, respectively (91.4 vs. 94.9 vs. 94.3%, *P* < 0.05). In the preschool group, most of the patients were girls (51.4%), while in the school-age group and the adolescent group, most of the patients were boys (*P* < 0.05). Among the children (*n* = 3,205) who underwent echocardiography, 108 children had positive results, and 3,097 children had negative results. Among the 108 positive results, 10 cases of cardiac diseases were related to chest pain. The sensitivity, specificity of echocardiography in the diagnosis of cardiac chest pain were 6.7, and 96.9%, while the positive predictive value and negative predictive value was 12.96 and 93.67%, respectively.

**Conclusion:**

In children with chest pain who are admitted to pediatric cardiology clinics, chest pain is mostly benign and rarely due to cardiac diseases. The use of echocardiography in evaluating cardiac chest pain in children is of little diagnostic value and leads to excess costs for patients and the health care system.

## Introduction

Chest pain is common not only in cardiology clinics, but also in other pediatric clinics. Different from the disease spectrum causing adult chest pain, children with chest pain always are benign, and very few are caused by cardiogenic diseases (1–5). For parents, when they hear their child describe chest pain, most parents fear that their child might have a cardiac disease and take them to a pediatric cardiology clinic to undergo a variety of auxiliary cardiac examinations, which brings spiritual and economic burdens to the child and their family. Therefore, the challenge for doctors is to identify benign chest pain from cardiac disease.

To date, few studies have reported the disease spectrum of chest pain in children who are referred to cardiology clinics (1, 6). Studies have estimated that cardiac chest pain in children range from 1 to 8% (7–10). In the existing studies, the number of subjects was small.

For children with chest pain as a chief complaint, doctors often let them undergo many auxiliary cardiac examinations to exclude cardiac disease when they are admitted to the cardiac clinic, such as electrocardiogram (ECG), echocardiography and Holter monitoring. Echocardiography is a non-invasive auxiliary examination which was used to assessment the structure and function of the heart and is widely used in cardiology clinics. Therefore, is echocardiography of high diagnostic value in assessing the etiology of cardiogenic chest pain in children? To date, no literature has been reported.

This study analyzed the disease spectrum of children’s chest pain in pediatric cardiac clinics and evaluated the diagnostic value of echocardiography in screening children with cardiac chest pain.

## Materials and Methods

### Case Enrollment

The clinical data of children with chest pain who admitted to pediatric cardiology clinic of Beijing Anzhen Hospital from 2005 to 2019 were analyzed. The disease was coded according to the 10th revision of the International Statistical Classification of Diseases and Related Health Problems (ICD-10). The exclusion criteria included that the clinical data of patients was incomplete. The study was approved by the Institutional Review Board oft Beijing Anzhen Hospital.

The demographics, history of the present illness, past medical history, family history, and auxiliary exams of these patients were analyzed. According to the above content and results, we diagnosed the etiology of children with chest pain. And then based on the clinical diagnoses, we determined the disease spectrum of chest pain in children who were admitted to the pediatric cardiology clinic. Based on the cause of chest pain, we divided the patients into the cardiac chest pain group and the non-cardiac chest pain group. According to the age of the patient, we divided these patients into three groups, including the preschool group (3 years ≤ age ≤ 6 years), the school-age group (6 years < age ≤ 12 years), and the adolescent group (12 years < age ≤ 18 years). The sex distribution and the etiology of chest pain in different groups were analyzed. Patients with congenital adrenal insufficiency, systemic lupus erythematous, carnitine deficiency or juvenile rheumatoid arthritis were thought to have positive history of past illnesses. If the family member of the patients which had been diagnosed with pulmonary arterial hypertension, Marfan syndrome, cardiomyopathy, severe familial hypercholesterolemia or had sudden death, we thought the family history was positive.

### Analysis of Clinical Auxiliary Exams and Diagnoses

ECG reports were retrieved from the clinical medical records. Abnormal ECG showed pathological Q wave, deviation of the electrical axis, atrial enlargement, pathological ST-segment or T-wave changes, pre-excitation syndrome, high-grade atrioventricular block, frequent premature ventricular contractions, ventricular hypertrophy and supraventricular tachycardia.

The results of other auxiliary exams including myocardial enzyme, echocardiography, chest X-ray and Holter monitoring results were analyzed according to the clinical reports.

Until now, children with myocarditis, pericarditis, large ventricular septal defect, anomalous origin of the coronary artery, cardiomyopathy, pulmonary arterial hypertension, aortic dissection, moderate or severe left ventricular outflow tract obstruction, pulmonary embolism or arrhythmia were thought to have cardiac chest pain (1–5, 11–16). According to our previous study, we enrolled suspected myocarditis as cardiac chest pain in children (10, 17). Based on criteria that were applied in retrospect, chest pain was divided into cardiac or non-cardiac (1–5, 13–17). According to the history of the present illness, past medical history, family history, and auxiliary exams of these patients, if he/she met the above diagnostic criteria of cardiogenic chest pain related diseases, we diagnosed him/her as cardiogenic chest pain.

### Diagnostic Value of Echocardiography for Cardiac Chest Pain

Children with chest pain without a history of cardiogenic diseases were divided into the echocardiography group and the non-echocardiography group according to whether they underwent echocardiography. The etiology of children in the echocardiography group was analyzed, and the diagnostic value for cardiogenic chest pain was evaluated according to the results of echocardiography.

### Statistical Analysis

For statistical analysis, SPSS 20.0 was used. Count data are expressed as the frequency (percentage), and measurement data are expressed as the mean ± standard deviation (*x̄* ± SD). The χ*^2^* test was performed to analyze the sex distribution and causes of chest pain. For the cardiogenic chest pain cohort, we calculated the sensitivity, specificity, positive and negative predictive values, and positive and negative likelihood ratios for echocardiography. The 95% CI for the sensitivity and specificity was computed by the Wilson method. A *P* < 0.05 was considered statistically significant.

## Results

**FIGURE 1:**
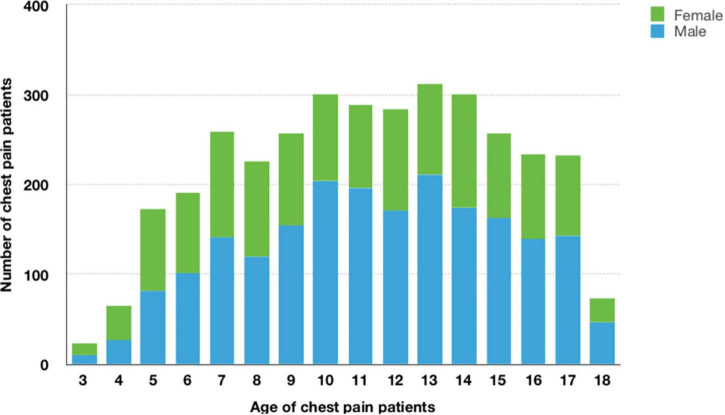
Frequency histogram of age and sex among patients with chest pain (*n* = 3,477). Of all 3,477 patients with chest pain, 2,083 (59.9%)were male, 1,394 (40.1%) were female, mean age was 11.2 ± 3.8 years.

In our study, 3,477 patients aged from three to eighteen years (11.2 ± 3.8 years) reported a chief complaint of chest pain, including 2,083 boys (59.9%) and 1,394 girls (40.1%). Girls (51.4%) were more common in the preschool group, while boys were more common in the school-age group (61.1%) and adolescent group (62.2%), and the sex distribution difference in the three groups was significant (*P* < 0.05) (Figure 1 and Table 1).

**TABLE 1:** The sex distribution of chest pain in children with different ages.

	*n*	3 years ≤ age ≤ 6 years	6 years < age ≤ 12 years	12 years < age ≤ 18 years
Total	3,477	451	1,615	1,411
Age (years)	11.2 ± 3.8	5.2 ± 0.9	9.6 ± 1.7	15.0 ± 1.5
Male	2,083 (59.9%)	219 (48.6%)	987 (61.1%)	877 (62.2%)
Female	1394 (40.1%)	232 (51.4%)	628 (38.9%)	534 (37.8%)
*X* ^2^			22.873	26.089
*P*			0.000*	0.000*

*Stands for P < 0.05 contrast to 3 years ≤ age ≤ 6 years group.

**FIGURE 2:**
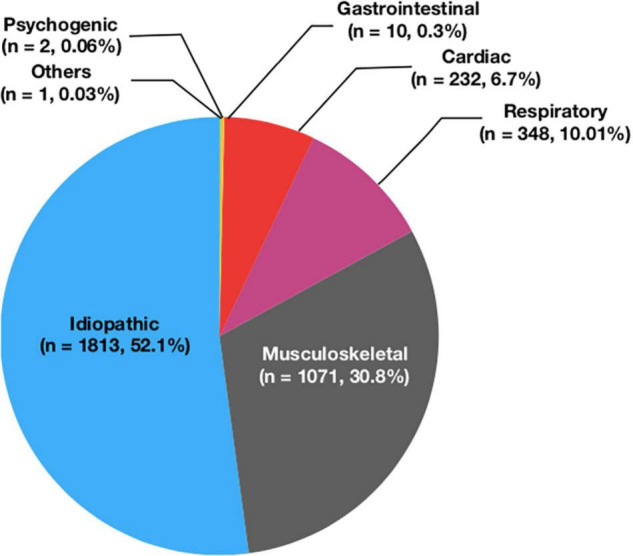
The disease composition of chest pain in children admitted to cardiac clinic.

232 patients (6.7%) were diagnosed as cardiac chest pain, and 3,245 patients (93.3%) were diagnosed as non-cardiac chest pain. In children with non-cardiac chest pain, 1,813 cases (52.1%) were idiopathic (unknown cause), 1,071 (30.8%) cases were skeletomuscular diseases, 348 (10.01%) cases were respiratory diseases, ten cases (0.3%) were gastrointestinal diseases, two cases (0.06%) were mental diseases, and one case (0.03%) was other condition (Figure 2). In the preschool group and the school-age group, the incidence of non-cardiac chest pain was significantly higher than it in the adolescent group (94.9 vs. 94.3 vs. 91.4%, respectively, *P* < 0.05) (Table 2).

**TABLE 2:** The cause of chest pain in children with different ages.

	*n*	3 years ≤ age ≤ 6 years	6 years < age ≤ 12 years	12 years < age ≤ 18 years
Total	3,477	451	1,615	1,411
Cardiac	232 (6.7%)	23 (5.1%)	92 (5.7%)	122 (8.6%)
Non-cardiac	3,245 (93.3%)	428 (94.9%)	1,523 (94.3%)	1,289 (91.4%)
*X* ^2^		5.986	9.970	
*P*		0.014^#^	0.002^#^	

*^#^Stands for P < 0.05 contrast to 12 years < age ≤ 18 years group.*

In 232 patients with cardiac chest pain, suspected myocarditis was the most common cause (*n* = 166, 71.6%), followed by myocarditis (*n* = 46, 19.8%), pulmonary arterial hypertension (*n* = 6, 2.6%), Kawasaki disease (*n* = 5, 2.2%), frequent premature ventricular contractions (*n* = 4, 1.7%), dilated cardiomyopathy (*n* = 2, 0.9%), pre-excitation syndrome (*n* = 1, 0.4%), anomalous origin of the left coronary artery (*n* = 1, 0.4%), and coronary-pulmonary arterial fistula (*n* = 1, 0.4%).

In 1,071 patients with skeletomuscular diseases, precordial catch syndrome was the most common cause (*n* = 802, 74.9%), followed by slipping rib syndrome (*n* = 262, 24.4%), costochondritis (*n* = 6, 0.6%), and straight-back syndrome (*n* = 1, 0.1%).

In 348 patients with respiratory diseases, bronchitis was the most common disease (*n* = 230, 66.1%), followed by pneumonia (*n* = 105, 30.1%), acute asthmatic bronchitis (*n* = 6, 1.7%), asthma (*n* = 2, 0.6%), pleuritis (*n* = 2, 0.6%), chronic cough (*n* = 1, 0.3%), emphysema (*n* = 1, 0.3%), and pulmonary tuberculosis (*n* = 1, 0.3%).

In ten patients with gastrointestinal diseases, gastroesophageal reflux was the most common cause (*n* = 5, 50.0%), followed by gastritis (*n* = 3, 30.0%), esophagitis (*n* = 1, 10.0%), and constipation (*n* = 1, 10.0%).

In two patients with mental diseases, one was anxiety and the other was depression. One case of chest pain due to other diseases was caused by poor postoperative wound healing.

Among all patients, 31 children had a history of cardiogenic diseases, of which 30 had undergone atrial septal defect repair and 1 had pre-excitation syndrome. A total of 3,346 children had no history of cardiogenic diseases, 3,205 children underwent echocardiography at least once, and 241 children had not undergone echocardiography. By consulting the outpatient doctors of pediatric cardiology department, we found that there were two reasons why patients did not undergo echocardiography. First, the patient’s parents or guardians refused to perform the examination. Second, according to the patient’s medical history, clinical manifestations and physical examination results, the doctor thought that the patient’s chest pain was not related to cardiogenic diseases, so the examination was not necessary.

**FIGURE 3:**
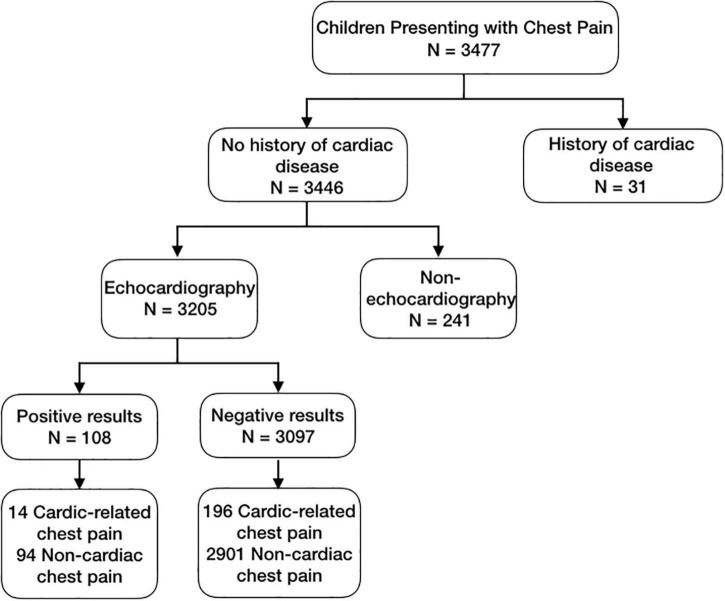
Profile of screening cardiac chest pain study by echocardiography.

Among the children who underwent echocardiography, 108 children had positive results, and 3,097 children had negative results. Among the 108 children with positive results, 10 children had chest pain due to cardiac diseases. Among the 3,097 children with negative results, 196 children had chest pain due to cardiac diseases (Figure 3). The sensitivity, specificity, positive predictive value and negative predictive value of echocardiography in the diagnosis of cardiac chest pain were 6.7, 96.9, 12.96 and 93.67%, respectively (Table 3).

**TABLE 3:** Accuracy of echocardiography for detection of cardiac cheat pain in children with chest pain.

	Echocardiography
True positive	14
False negative	196
False positive	94
True negative	2901
False-positive rate	3.1%
Sensitivity (95% CI)	6.7% (4.0–10.9)
Specificity (95% CI)	96.9% (96.2–97.5)
Positive predictive value (95% CI)	12.96% (7.88–20.59)
Negative predictive value (95% CI)	93.67% (92.76–94.47)
Positive likelihood ratio (95% CI)	2.16% (1.23–3.66)
Negative likelihood ratio (95% CI)	0.96% (0.93–1.00)

## Discussion

Chest pain is a common symptom in children, and the visit rate of chest pain as the first complaint in pediatric outpatient is gradually increasing (10, 18). Since there is not enough relevant knowledge about the common causes of children’s chest pain, most parents believe that their child’s chest pain should be caused by cardiac diseases and may be life-threatening. Therefore, when a child complains of chest pain, their parents will take them to an emergency department or a pediatric cardiology clinic as soon as possible. In 2011, Hanson CL analyzed the medical records of patients with chest pain admitted to the University of Wisconsin Children’s Hospital between 2004 and 2006 and found that only 1 patient (0.7%) with chest pain was finally confirmed with a cardiac cause (6). A systemic review revealed that only 1.2% of patients with chest pain admitted to a major pediatric cardiology center had cardiac diseases (19). In 2011, Saleeb SF analyzed the incidence of sudden cardiac death in patients with presumed non-cardiac chest pain discharged from a cardiology clinic. They found that it’s common of chest pain in children, however, chest pain due to cardiac etiology is rare. Furthermore, after analyzing the entire data of cardiology visits over 1 decade (nearly 18,000 patient years), they found that there was no one patient discharged from the clinic died of a cardiac condition (18). To our knowledge, till now, there was no large sample report on the etiology and disease spectrum of chest pain in children referred to pediatric cardiology clinics in China. Clarifying the etiology and disease spectrum of Chinese children’s chest pain in pediatric cardiology clinics is of certain significance for the dissemination of knowledge related to children’s chest pain and alleviating the fear of children and their parents.

In our study, we analyzed 3,477 children with chest pain admitted to the pediatric cardiology clinic of Beijing Anzhen Hospital over the past 15 years. It was found that 6.7% of the children had cardiac diseases, and there was significantly more chest pain caused by non-cardiac diseases in preschool and school-age children than in adolescent children. The most common non-cardiac disease causing chest pain in children was idiopathic chest pain (52.1%), followed by musculoskeletal diseases (30.8%),respiratory diseases (10.01%),gastrointestinal diseases (0.3%), psychogenic diseases (0.06%) and other diseases (0.03%). In our study, the proportion of cardiac diseases in children as the cause of chest pain was only 6.7%, extremely low compared with other causes. Our result was consistent with Hanson CL’s, which showed that only one patient (0.7%) had a cardiac cause in children with chest pain admitted to the pediatric cardiology clinic (6). Similarly, Squires D et al. also reported that only 1.2% of patients with chest pain presenting to a major pediatric cardiology center had identifiable cardiac causes (19). The proportion of cardiac causes in children with chest pain admitted to our pediatric cardiology clinic was higher than that reported in the above-mentioned literature, which might be because that we included the suspected myocarditis in the category of cardiac chest pain. In our study, the proportion of psychogenic diseases in children with chest pain was lower than that reported in other literatures (6, 18). This may be related to the following reasons. This study was a retrospective study. We could only diagnose children who had recorded psychiatric evaluation as psychogenic diseases. Children without psychiatric evaluation records might be included in idiopathic chest pain, which might eventually lead to a low proportion of psychogenic diseases.

About the demographic characteristics of children with chest pain, some literatures have reported that female children with a mean age of about 10 years are often have chest pain (4, 20). Other studies showed that children with age over 12 years and in puberty are prone to chest pain (5, 6, 16). In 2020, Yoldaş T. et al. (21) studied the relationship between non-cardiac chest pain and internalizing problems in pre-school aged children, they found that these children might be related to increasing levels of some behavioral comorbidities. In 2020, Aygun E et al. (22) analyzed the etiology of 782 patients aged between 3 and 18 years with chest pain, they found that male patients were more prone to musculoskeletal and gastrointestinal systems diseases, while female patients were more prone to psychogenic disorders. In our study, girls (51.4%) were more common in the preschool group, while boys were more common in the school-age group (61.1%) and adolescent group (62.2%). This result is somewhat different from that reported in the above-mentioned literatures (4, 20), which might be because that this study was a single center study, the patients’ demographic characteristics in our study may be have some limitations. In our study, in the preschool group and the school-age group, the incidence of non-cardiac chest pain was significantly higher than it in the adolescent group, respectively. This might be related to increasing levels of some behavioral comorbidities as described above (21). For clarifying the effects of demographic characteristics on the distribution of chest pain etiology, we will conduct prospective, multi-center and social psychology comprehensive research on children with chest pain in the future.

Echocardiography is one of the most widely used, safe, non-invasive and cost-effective auxiliary examination in cardiovascular medicine (23). Most of cardiac diseases could be accurately evaluated by echocardiography, including such as cardiomyopathy, pericardial effusion, valvular heart disease, congestive heart failure, cardiac malformation, bacterial endocarditis, and so on (24, 25). Transthoracic echocardiography is a preferable auxiliary examination because it is rapidly and widely available, feasible for bedside use, non-invasive and cost-effective for patients with chest pain admitted to an emergency department (26). Some researchers regarded echocardiography as a “biomarker,” because it can provide information on anatomy, function and hemodynamics of heart (24, 25). Meanwhile, since in emergency and intensive care medicine, echocardiography could provide important information for many cardiovascular diseases, such as heart failure, acute coronary syndrome, pulmonary embolism, myocardial infarction, sepsis, stroke, endocarditis, and so on, some researchers recommended that all patients with chest pain must receive echocardiography examination (27). However, another research conducted by Gibbons RJ et al. (28) showed that echocardiography examination was not necessary in chest patients due to its low positive detection rate. In their research including all referral outpatients’ data from January 2010 to December 2013 in Mayo Clinic Rochester, they aimed to identify patients with stable chest pain and known or suspected coronary artery disease, all patients had normal resting ECGs and received resting echocardiography examination. The result indicated that unnecessary echocardiograms were normal and had little impact on clinical treatment. Therefore, they advised that unnecessary echocardiograms could be avoided (28). Furthermore, it is still controversial whether echocardiography is needed to evaluate the etiology of chest pain.

In clinical practice, owing to echocardiography’s availability and non-invasive nature, it was often used by pediatric cardiologists to evaluate chest pain in children. Over the past 20 years, the application of echocardiography examination has grown significantly in both pediatric and adult settings, this trend is in accordance with an increase in the use of all diagnostic imaging in the United States (29–32). In 2017, Chamberlain RC (33) et al. evaluated the diagnostic yield and frequency of echocardiograms which were performed for each “appropriate use” indication criteria, they found that for these patients, echocardiogram use was of little diagnostic utility. On the other hand, they evaluated the cost related to echocardiograms performed for indications meeting the “rarely appropriate” criteria. The result showed that in patients meeting the “rarely appropriate” indication criteria, echocardiogram use contributed to additional costs for the patients and health care system (33). However, until now, it is still not clear of the diagnostic value of echocardiography for evaluating cardiac chest pain in children.

Among the patients we enrolled in the study, 31 patients had a history of cardiac disease. In 3,446 patients with no history of cardiac disease, 3,205 patients underwent echocardiography. Based on the results of echocardiography, we found that the sensitivity, specificity, positive predictive value and negative predictive value of echocardiography for the diagnosis of cardiac chest pain were 6.7, 96.9, 12.96, and 93.67%, respectively. The sensitivity and positive predictive value are low, and the specificity and negative predictive value are high, indicating that the use of echocardiography in evaluating cardiac chest pain in children is of little diagnostic utility and contributes to additional costs for patients and the health care system. This is basically consistent with previous literature reports (33). Therefore, for the evaluation of children’s cardiac chest pain, a comprehensive analysis should be performed according to a child’s clinical manifestations, past history, family history and physical examination and an appropriate auxiliary examination should then be selected to identify and clarify the cause, which can not only save clinical resources but also reduce the economic burden on children and their families.

In this large retrospective study, we found that among preschool children with chest pain, girls were dominant, while for school-age children and adolescents, chest pain is more common in boys. The most common cause of chest pain in Chinese children admitted to pediatric cardiology clinic is idiopathic chest pain, and very few children suffer from chest pain caused by cardiac diseases. Echocardiography has low sensitivity and positive predictive value for the evaluation of cardiac chest pain in children, and it is of little diagnostic utility and contributes to additional costs for patients and the health care system. For the evaluation of children’s cardiac chest pain, a comprehensive analysis should be performed according to a child’s clinical manifestations, past medical history, physical examination, an appropriate auxiliary examination and family history should then be selected to identify and clarify the cause, which can not only save clinical resources but also reduce the economic burden on children and their families.

The limitations of this study including: (1) This study was a single center study, which only represents the case composition of a single center. And the relevant data in this study might be affected by the hospital size, patients’ source, first line specialty and other aspects. In the future, to more accurately evaluate the disease composition spectrum of chest pain in Chinese children who are treated in pediatric cardiology clinics, multicenter and large sample research studies should be conducted for analysis. (2) This study was a retrospective study. Some data were missing, which may affect some results. In the future, we will perform further prospective research to clarify the reasonable application time of echocardiography in the diagnosis of cardiac chest pain in children.

## Data Availability Statement

The original contributions presented in the study are included in the article/supplementary material, further inquiries can be directed to the corresponding author/s.

## Ethics Statement

The studies involving human participants were reviewed and approved by Beijing Anzhen Hospital Ethics Committee. Written informed consent to participate in this study was provided by the participants’ legal guardian/next of kin.

## Author Contributions

HD, LC, and GL conceptualized the research hypothesis and analyses. LC researched the data, performed all of the statistical analyses, and wrote the manuscript. XL reviewed and edited the manuscript. GL obtained informed consent and checked manuscript. All authors read and approved the final manuscript.

## Conflict of Interest

The authors declare that the research was conducted in the absence of any commercial or financial relationships that could be construed as a potential conflict of interest.

## Publisher’s Note

All claims expressed in this article are solely those of the authors and do not necessarily represent those of their affiliated organizations, or those of the publisher, the editors and the reviewers. Any product that may be evaluated in this article, or claim that may be made by its manufacturer, is not guaranteed or endorsed by the publisher.
